# An Update on Gastrointestinal Parasitic Infection in Captive Wild Animals in Bangladesh

**DOI:** 10.1155/2023/3692471

**Published:** 2023-12-28

**Authors:** Rehana Rahman, Jannatul Nyema, Md. Imranuzzaman, Bijoy Banik, Proshanto Singha Pranto, Kanan Talukder, Susmita Rani Sarkar, Shampa Deb Nath, Kazi Mehetazul Islam, Tilak Chandra Nath, Saiful Islam

**Affiliations:** ^1^Faculty of Veterinary, Animal and Biomedical Sciences, Sylhet Agricultural University, Sylhet 3100, Bangladesh; ^2^Department of Parasitology, Sylhet Agricultural University, Sylhet 3100, Bangladesh; ^3^Department of Pharmacology and Toxicology, Sylhet Agricultural University, Sylhet 3100, Bangladesh; ^4^Department of Animal Nutrition, Sylhet Agricultural University, Sylhet 3100, Bangladesh; ^5^Department of Pathology, Sylhet Agricultural University, Sylhet 3100, Bangladesh

## Abstract

Gastrointestinal parasites cause health problems and even death in captive animals. These animals are more susceptible to parasitic infestations because of confinement and stress. The aim of the study is to describe parasitic causal agents in captive wild animals at the Bangladesh National Zoo, Chittagong Zoo, and Tilagarh Eco Park, in Sylhet. A total of 54 fecal samples were collected from the Bangladesh Zoo, Chittagong Zoo, and Tilagarh Eco Park from April 2022 to November 2022. Samples were collected from animals of the groups Aves (16), Reptiles (4), Artiodactyla (23), Perissodactyla (8), and Proboscidea (3). All samples were examined using a modified formalin ether sedimentation technique. Fecal examination consoled an overall occurrence of 61.1%. Out of 54 samples, 33 were positive for parasitic infection. The parasites observed were as follows: Ascarididae eggs (57.58%), *Capillaria* spp. (18.18%), *Strongyloides* spp. (9.09%), *Trichuris* spp. (6.06%), and mixed infection (9.09%). The order Reptiles had a 100% infection rate, while Aves had the lowest infection rate (50%). Only nematodes were detected in this study. Without showing evident, clinical signs and symptoms of disease, the prevalence of gastrointestinal parasites is high. This indicates a subclinical infection. This study shows that more epidemiological research and sanitation management programs, including regular antihelminthic therapy for controlling parasitic infection, should be adopted in zoos and ecoparks.

## 1. Introduction

Zoos serve as *ex situ* conservation facilities for wild animals. These places are crucial for the protection of endangered species. Zoos engage in species conservation as well as research and environmental education. In terms of that, controlling infectious diseases and maintaining proper husbandry are vital things for keeping wild animals in captivity [1]. In the wild, animals have some natural resistance to parasitic infections, and the parasite and host are typically in an equilibrium condition that sometimes results in a very serious infection [2]. Animals living in cramped cages and constrained surroundings are stressed; their immune systems weaken, leaving them more susceptible to parasite illnesses [1]. In the case of captivity, the health of zoo animals depends on many factors. Examples include management, dietary habits, the environment, cleanliness, and seasonal changes. Several researchers have documented the presence of numerous parasites in caged wild animals [1, 3]. However, some gastrointestinal parasites of wild animal may endanger for the health of zoo staff, tourists, and city residents. Because many gastrointestinal parasites can infect several host species. Knowledge of the illness states of zoo animals and appropriate screening are important for public health safety and animal welfare [1]. Despite the fact that gastrointestinal parasites have been found in both wild and captive animals worldwide [4], unquestionably, the health of zoo animals will be improved by a continuous program of gastrointestinal parasite surveillance and control approaches, including efficient treatment and sufficient prophylactic based on precise diagnosis [5].

The study of wild animal diseases is significant for comparative pathology, public health, and medicine. Diseases of wild animals in aberrant hosts can cause epidemics with considerable mortality [6]. Globally, wildlife has been found to be a significant source of new zoonotic illnesses, and the majority of emerging and reemerging infections originated in animal reservoirs [7]. By definition, zoological gardens gather a variety of animal species in close proximity, and many of these animals are unfamiliar with their geographical region. Close proximity allows for disease or parasite transfer to species that would not ordinarily come into contact with these pathogens.

In this situation, transmission can occur from exotic to indigenous animals or vice versa; usually, animals with no prior interaction with the pathogens are very susceptible to infection [6]. Recent studies have shown that animal reservoirs can be formed through parasite infections transferred directly from human hosts. The idea that parasites in humans are a natural reservoir that can spread directly to animals is not widely accepted, nor is the fact that when this happens, a new reservoir of potential public health relevance may be generated in wildlife [8]. The most prevalent gastrointestinal parasites of confined animals in Bangladesh include *Ascaris* spp., *Capillaria* spp., *Strongyloides* spp., *Trichuris* spp., and *Moniezia* spp. [9, 10]. Only a few studies have been done in zoos to detect gastrointestinal parasitic illnesses, despite the fact that it is vital to understand the transmission and zoonotic potentiality of existing parasites inside the wild animals of zoological parks. Due to this, the current study focused on how commonly gastrointestinal parasites occur in Bangladeshi confined animals.

## 2. Materials and Methods

### 2.1. Study Area and Time

**Figure 1:**
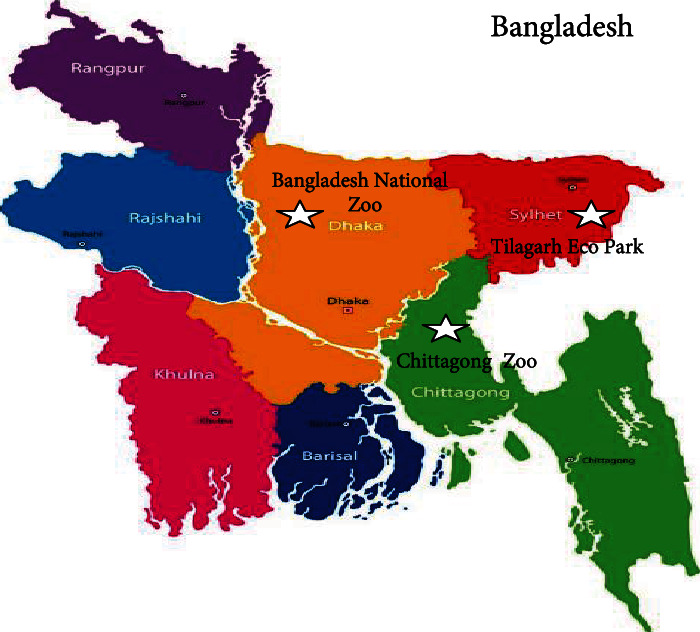
Study area (star marks indicating sample collection locations).

This study was conducted at the Chittagong Zoo, the Tilagarh Eco Park, and the Bangladesh National Zoo in Dhaka, Sylhet, and Dhaka, respectively.  Figure 1 shows these research locations in Bangladesh. The samples were evaluated in the Sylhet Agricultural University's Parasitology lab. The research was carried out between April 2022 and November 2022.

### 2.2. Study Populations

A total of 54 fecal samples were collected from different species, including *Axis axis* (14), *Bos frontalis* (3), *Camelus dromedarius* (3), *Elephas maximus* (3), *Equus ferus caballus* (2), *Equus quagga* (4), *Equus asinus* (2), *Taurotragus oryx* (3), *Ara ararauna* (3), *Pavo cristatus* (7), *Psittacus erithacus* (3), *Lophura nycthemera* (3), and *Python molurus* (4).

### 2.3. Gathering and Preserving Fecal Samples

To minimize contamination, fecal samples were collected from the individual animal cage in the early morning with the assistance of an animal caretaker. Each sample was placed in a plastic container containing 10% formalin. The containers were kept in plastic biohazard bags to transport the samples. The samples were labeled according to species with a marker, and the opening edge of the bag was tightly closed.

### 2.4. Examination of Fecal Samples and Analysis of Data

All samples were evaluated at the parasitology laboratory of Sylhet Agricultural University, Sylhet. A microscopic inspection of the samples was prepared. Each sample was examined by a modified formalin ether sedimentation technique [1], and eggs were identified by following Soulsby [11]. The information was analyzed using STATA 13.1 data analysis software. Descriptive statistics was used to tabulate and summarize the data.

## 3. Results

### 3.1. Total Infection Rate of Gastrointestinal Parasites in Animals

A total of 54 fecal samples were examined, of which the overall occurrence of helminth infection was 61.11% (Table 1). The highest rate of helminth infection was found in Reptiles 100.00%, and the lowest rate was in Aves (50.00%). In Artiodactyla, Perissodactyla, and Proboscidea, the rates of gastrointestinal helminthic infection were 56.52%, 75.00%, and 66.67%, respectively. Mixed infection was found in 9.09% of animals. Results indicated that Ascarididae infection was more common than other helminth infections in zoo animals. In the current study, we found (100.00%) helminth infection in zebra, python, and macaw, whereas silver pheasants were free of any helminth infection.

### 3.2. Infection Frequency of Gastrointestinal Parasites in Herbivores

The percentage of herbivores with gastrointestinal helminths was 61.76%. The isolated helminths included Ascarididae eggs, *Capillaria* spp., *Strongyloides* spp., and *Trichuris* spp.

### 3.3. Infection Frequency of Gastrointestinal Parasites in Birds

Among birds, 50.00% of samples were positive for gastrointestinal helminths. The identified helminths were Ascarididae eggs (*Ascaridia/Heterakis*), *Capillaria* spp., *Strongyloides* spp., *Trichuris* spp.

### 3.4. Infection Frequency of Gastrointestinal Parasites in Reptiles

Among reptiles, 100% (4) of samples were positive for gastrointestinal helminths. The detected helminths were Ascarididae eggs (4/19, 21.05%).

## 4. Discussion

Zoological gardens are made to safeguard biodiversity and preserve animal species that are in danger of extinction. Learning about the numerous diseases that plague wild and exotic animals in captivity is beneficial [12]. Zoo animals, for instance, are parasitized by a variety of endoparasite species. In that circumstance, zoo management should prioritize parasite control since helminth infection has a negative influence on the health of caged animals [13]. More than half of the investigated animals were found to be infected with at least one helminth species, and the study detected the eggs/larvae of 4 distinct types of parasites. Since 12 out of the 13 species that were studied were coprologically positive, the current data demonstrate that parasites can be widespread among zoo animals. It should be noted that while the results are intriguing, they are more experiential than statistical because the study only used a small number of animals.

In all, 61.11% of the animals at the Bangladesh National Zoo, Chittagong Zoo, and Tilagarh Eco Park were discovered to be infected with GI helminths during the course of our investigation, which was close to a previous finding (60.00%) made by Khatun et al. [9] at the Rangpur Recreational Garden and Zoo in Bangladesh. Our findings conflict with earlier studies by Opara et al. [14] and Corden et al. [15] in Nigeria (76.60%) and Spain (72.50%) and also with the studies on the prevalence of gastrointestinal parasites in the Dhaka Zoological Garden that found gastrointestinal parasite prevalence to be higher at 76.95% [16] and 78.60% [10], respectively. The present study was also dissimilar to the prevalence of gastrointestinal helminths reported by Thawait et al. [17] (46.20%) and Nath et al. [1] (46.50%); however, the prevalence ranged consistently between 46.20% and 76.95%. The research area's climate, husbandry practices, and food management might all be contributing factors to the difference.

Employees of zoos have reportedly also contributed to transmission by serving as vectors and dispersing parasites through their footwear, attire, hands, food, and equipment. According to the reports of Adetunji [4] as well as Otegbade and Morenikeji [18], zoo employees may contribute to environmental contamination through tainted water or feed. The intense husbandry of wild animals in zoos and zoological parks may be one of the reasons for greater infection because of the high animal density in enclosures and their proximity to different species of animals, which allows for parasite transmission [2]. This finding highlights the significance of continuing to monitor and treat these animals in order to improve management practices [19]. The occurrence of gastrointestinal parasites in Artiodactyla and Perissodactyla in a previous study recorded by Barbosa et al. [20] was 33.3% and 100%, respectively, where our study found a prevalence rate of 56.5% and 75%, respectively.

In this study, it was revealed that 61.76% of herbivores were infected. Wahed [21] (44.4%), Nath et al. [1] (36.9%), and Khatun et al. [9] (30.8%) all found lower results than this one. In this study, 57.4% of the spotted deer tested positive for gastrointestinal parasites, which is greater than the prevalence reported by Khatun et al. [9] (43.5%) and lower than the prevalence seen by Kanungo et al. [22] (75%). According to the data, 50% of the birds tested positive for GI illness. The prevalence was reported by Hossain et al. [10] and Nath et al. [1] to be 42.9% and 40%, respectively. This finding is lower than those reports. Geographical and environmental factors are the reason for this variation. The silver pheasants showed no symptoms of gastrointestinal helminth. This can be due to the sample size, deworming, and feeding management. In the current investigation, 100% of reptiles were positive for GI parasites. The placement of the animal cages, the presence of intermediate hosts nearby the cages, and the feed supply might all be contributing factors to the high infection rate. Zoo reptiles may be infected with zoonotic parasites, and they regularly pass away from gastrointestinal parasites that go unnoticed. In order to prevent the spread of disease to humans and to save the reptiles' lives, it is crucial to screen reptiles before putting them in zoological gardens and to treat positive results as soon as possible [19]. The presence of parasitic nematodes belonging to the family Ascarididae in birds has long been a topic of interest, with potentially significant implications for the health and ecology of both the parasites and their avian hosts.

Spotted deer and macaws both had mixed infections in the current investigation. Kanungo et al. [22] also noted that the majority of the deer had mixed illness. The fact that different age groups of animals were housed in the same cages, poor feeding practices, and inappropriate excrement disposal could all have contributed to the study's mixed infection finding. Between the prior study and our current investigation, there were significant differences in the mixed infection rate. The rate was 48% in a prior study [2]; however, it was about 9.09% in our study. There are a few restrictions because the goal of our experiment was to create a baseline inventory of important endoparasite species infecting confined animals in Bangladesh. We did not set out to provide in-depth analyses of every species or to identify them at the species level.

Gastrointestinal parasites can infect captive animals within zoo environments due to factors such as restricted living spaces, limited access to natural foraging, and stress associated with captivity, which can compromise the animals' immune systems. This susceptibility poses health risks to captive animals, including nutritional deficiencies, digestive disorders, and weakened immunity. Concurrently, zoo employees and visitors face potential exposure risks, as certain gastrointestinal parasites may have zoonotic potential. To control these parasites, comprehensive measures are crucial. These include regular veterinary monitoring, implementing parasite prevention programs through the administration of anthelmintic medications, optimizing animal diets to support immune health, maintaining clean living conditions, and employing proper hygiene protocols for both animal care personnel and visitors. By integrating these measures, zoos are aimed at safeguarding the health and well-being of both captive animals and those interacting with them.

The study has several limitations. One significant limitation of this study is the relatively small sample size of captive wild animals in Bangladesh that were included in the research. Due to logistical constraints, the sample size may not fully represent the country's entire population of captive wild animals. The animals included in this study were selected from specific captive facilities, and there might be inherent biases in the selection process. This study did not delve deeply into species-specific variations, which could be essential for understanding the dynamics of parasitic infections in captive wild animals. The study primarily relied on traditional parasitological methods, such as fecal examinations, for identifying parasites. The use of molecular techniques for species-level identification of parasites could provide more detailed insights into the types of parasites present. A more comprehensive investigation involving a wider range of species would provide a more complete picture of the prevalence and diversity of gastrointestinal parasites.

## 5. Conclusion

Zoo animals serve as both hosts and reservoirs for a variety of zoonotic parasites. To promote conservation, education, and aesthetics, zoological parks display wild animals (including fish, birds, reptiles, amphibians, and mammals). The animals seemed to be healthy during the examination period, and no reported deaths or clinical symptoms were noted; nonetheless, the high incidence suggests subclinical infection that may induce disruption in stressful situations and produce pathogenicity. Species and management have an impact on how frequently wild animals kept in captivity become parasitized. More research on parasitic infections is necessary to comprehend the epidemiology of parasitism prevention and to improve parasite infection prevention. Our preliminary observations may help us identify some of the important parasitic species affecting captive animals in Bangladesh.

## Figures and Tables

**Table 1:** Occurrence of gastrointestinal parasites in captive wild animals from three zoos in Bangladesh.

Type of host	Host	Scientific name	No. of examined samples	No. of positives (%)	Egg or larvae observed (helminths)				
					Ascarididae eggs	*Capillaria* spp.	*Strongyloides* spp.	*Trichuris* sp.	Mixed infection
Mammals	Spotted deer	*Axis axis*	14	8 (57.14)	4	2	—	—	2
	Gayal	*Bos frontalis*	3	2 (66.67)	2	—	—	—	—
	Camel	*Camelus dromedarius*	3	2 (66.67)	2	—	—	—	—
	Asian elephant	*Elephas maximus*	3	2 (66.67)	2	—	—	—	—
	Arabian horse	*Equus ferus caballus*	2	1 (50.00)	—	—	1	—	—
	Zebra	*Equus quagga*	4	4 (100.00)	4	—	—	—	—
	Donkey	*Equus asinus*	2	1 (50.00)	—	—	1	—	—
	Eland	*Taurotragus oryx*	3	1 (33.33)	—	—	—	1	—
	8 host species		34	21 (61.76)		14	2	2	1

Birds	Macaw	*Ara macaw*	3	3 (100.00)		1	1		1
	Peacock	*Pavo cristatus*	7	3 (42.86)		2		1	
	Grey parrot	*Psittacus erithacus*	3	2 (66.67)	1	1			
	Silver pheasant	*Lophura nycthemera*	3	—	—	—	—	—	—
	4 host species		16	8 (50.00)		1	4	1	1

Reptiles	Python	*Python molurus*	4	4 (100.00)	4	—	—	—	—
Total	13 host species		54	33 (61.11)		19 (57.58%)	6 (18.18%)	3 (9.09%)	2 (6.06%)

## Data Availability

On an appropriate request, the corresponding author and the Department of Parasitology, Sylhet Agricultural University, Bangladesh, will provide the samples applied for the present research available.
